# Relationship of Omega-3 Fatty Acids on C-Reactive Protein and Homocysteine in Haitian and African Americans with and without Type 2 Diabetes

**DOI:** 10.4172/2155-9600.1000180

**Published:** 2012-12-19

**Authors:** Fatma G. Huffman, Joan A. Vaccaro, Joel C. Exebio, Sahar Ajabshir, Gustavo G. Zarini, Lemia H. Shaban

**Affiliations:** Department of Dietetics and Nutrition, Florida International University, USA

**Keywords:** n-3 fatty acids, Homocysteine, C-reactive protein, African Americans, Haitian Americans, Type 2 diabetes

## Abstract

**Background:**

Omega-3 fatty acids (n-3) may be protective of cardiovascular risk factors for vulnerable populations. The purpose of this study was to assess the association between n-3 with, C-reactive protein (CRP), and homocysteine (HCY) in Black minorities with and without type 2 diabetes.

**Methods:**

A cross-sectional study was conducted with 406 participants: Haitian Americans (HA): n=238. African Americans (AA): n=172. Participants were recruited from a randomly generated mailing lists, local diabetes educators, community health practitioners and advertisements from 2008–2010. Sociodemographics and anthropometrics were collected and used to adjust analyses. All dietary variables were collected using the semi-quantitative food frequency questionnaire (FFQ) and used to quantify vitamin components. Blood was collected to measure CVD risk factors (blood lipids, HCY, and CRP).

**Results:**

African Americans had higher waist circumferences and C-reactive protein and consumed more calories as compared to Haitian Americans. Omega 3 fatty acid intake per calorie did not differ between these ethnicities, yet African Americans with low n-3 intake were three times more likely to have high C-reactive protein as compared to their counterparts [OR=3. 32 (1. 11, 9. 26) p=0.031].

Although homocysteine did not differ by ethnicity, African Americans with low omega 3 intake (<1 g/day) were four times as likely to have high homocysteine (>12 mg/L) as compared to their counterparts, adjusting for confounders [OR=4.63 (1.59, 12.0) p=0.004]. Consumption of n-3 by diabetes status was not associated with C-reactive protein or homocysteine levels.

**Conclusions:**

Consumption of n-3 may be protective of cardiovascular risk factors such as C-reactive protein and homocysteine for certain ethnicities. Prospective studies are needed to confirm these results.

## Introduction

Cardiovascular disease (CVD) is one of the most prevalent causes of morbidity and mortality worldwide [1]. Since it is possible to have normal blood lipids and be at risk for CVD, other blood markers such as C-reactive protein (CRP) [2–3] and homocysteine (HCY) [4] have been considered independent risk factors for CVD. It has been well-established that CVD involves systematic inflammation [5]. Prospective studies and primary prevention trials have demonstrated that CRP, an acute phase protein and marker of chronic, low-level inflammation, predicts CVD risk as well as CVD in those without prior disease [6]. High blood levels of HCY are thought to play a role in endothelial damage through oxidative stress-based on molecular studies and have been established as a risk factor for CVD by retrospective and prospective studies [4].

Omega-3 fatty acids (n-3) have been associated with antioxidant properties related to the reduction of CVD risk in observational and randomized control trials [7]. In addition to their antioxidant properties, n-3 may be associated with reducing systemic inflammation. N-3, a type of polyunsaturated fat derived from either fish (as eicosapentaenoic acid (EPA) or docosahexaenoic acid (DHA) or plant sources, α-linolenic acid (ALA) have been shown to have a protective effect on cardiovascular disease (CVD) risk in numerous epidemiological studies [8–11]. Whether this effect is due to their antioxidant or anti-inflammatory capacity, it has not been clearly established.

Participants with diabetes are under increased oxidative stress due to factors such as: auto-oxidation of glucose which produces more free radical formation, imbalances in cellular oxidation and reduction, and decreased antioxidant defense systems [12]. Atherosclerosis and atherothrombosis processes are up-regulated in persons with diabetes [13]. Individuals with diabetes have more than double the risk for CVD as compared to their counterparts [14]. 90–95% of the cases of diabetes type 2 incidences (T2D), has been attributed to genetic predisposition and lifestyle factors such as diet, physical activity, alcohol, obesity, and smoking [14].

Blacks are among those at high risk for and a higher prevalence of diabetes (11.8%) which places them at greater risk for CVD [14]. There is limited research on T2D and CVD risk factors in Haitian Americans (HA) and African Americans (AA). The majority of the existing data groups Blacks together; hence, there are no clear distinctions and acknowledgement of the widespread differences in culture, foods, and lifestyle among Black subgroups. In addition, there is no research, to date, on the association between n-3 with CVD risk factors specific to Black populations with T2D. It is possible that ethnicity may modify the effect of n-3 on either or both HCY and CRP. Therefore, this study investigated the association of n-3 with biomarkers of CVD risk: HCY and CRP, in HA and AA with and without T2D. The following hypotheses were tested: Participants with low n-3 intake, regardless of ethnicity and diabetes status, will be more likely to have: 1) high CRP, and 2) high HCY. The establishment of cut-off points for n-3, CRP, and HCY were based on clinical significance and the sample medians, as explained in the Statistical analysis section.

## Methods

### Study participants

This was a cross-sectional study of AA and HA with and without T2D. AA was recruited from Miami-Dade and Broward counties in Florida, USA using mailing lists purchased from Knowledge Base Marketing, Inc. The subjects were randomly selected by alternating between subjects with and then without T2D. For AA, 7,550 letters were mailed to potential participants with and without T2D, four percent responded (n=256), and 6.3% (n=477) were returned due to unknown addresses. HA participants (n=259) were recruited differently because mailing lists were not available for this group. Recruitment was done in the community through local diabetes educators and community health practitioners in Miami-Dade and Broward Counties, who were either former students or in close contact with the Department of Dietetics and Nutrition at Florida International University (FIU). Also, FIU faculty, staff and students, as well as, several residential rental facilities received invitational flyers and were requested to assist with the recruitment process. Advertisements were circulated in local Haitian newspapers, churches, supermarkets, restaurants, and also aired on local Creole stations. Interested participants were interviewed on the phone to obtain age, gender, and T2D status. Exclusion criteria consisted of those being younger than 35 years old, other ethnicity, severe diabetes complication (blindness, amputations, kidney failure) and having other major chronic diseases or illnesses, specifically, liver disease, congestive heart failure, bariatric surgery, cancer, AIDS/HIV. Eligible subjects were requested to come to the Human Nutrition Laboratory at FIU for blood collection after an eight-hour overnight fast; participants were asked to refrain from smoking and engaging in any unusual exercise prior to this visit. The study was approved by the Institutional Review Board at FIU. Written consent in English or Creole was required from all participants; and, all participants signed an informed consent form prior to enrollment in the study.

### Data collection

Participants completed a socio-demographic questionnaire, which included: age, gender, duration of residence in the USA, language preference, education, income, employment status, medications, and family history of T2D and CHD. Weight, height, waist circumference and blood pressure were performed in the Human Nutrition Laboratory of the Principal Investigator. Body mass index (es) (BMI) was calculated as weight in kg/height in m^2^.

#### Blood sampling:

A certified phlebotomists collected a 20 ml sample of venous blood from each subject after an 8-hour overnight fast. A vacationer Serum Separator Tube (SST) was used for the lipid profile sample. After complete coagulation (i.e., 30–45 minutes), the SST was centrifuged at 2500 rpm for 30 minutes. One sample of the serum was used for lipid profile analysis and the other was frozen at −70°C for HCY and high-sensitivity C-reactive protein (hs-CRP) analysis. Laboratory results showed that 13 participants (HA=8; AA=4) who reported not having diabetes were reclassified as having T2D according to the American Diabetes Association Standards. These participants were referred to their physicians and were included in the group with T2D.

#### Biochemical analysis:

Plasma total cholesterol (TC), triglycerides (TG), low-density lipoprotein cholesterol (LDL-C), and high-density lipoprotein cholesterol (HDL-C) were assayed by enzymatic methods (Cobas Mira, Roche Diagnostics, Indianapolis, IN). LDL-C was estimated by the Friedewald formula: {LDL-Cl=TC (mmol/L)-HDL-C (mmol/L)-TG (mmol/L) 2.2 (mmol/L)}. Hs-CRP was analyzed by Immulite (Diagnostic Products Corporation, Los Angeles, CA). The combined free and protein-bound plasma HCY was measured using the IMx System based on the Fluorescence Polarization Immunoassay technology (Axis-Sheild ASA, Oslo, Norway; Abbot Diagnostic Division, Abbott Laboratories, Abbott Park, IL).

#### Nutritional variables:

All dietary variables were collected using the semi-quantitative food frequency questionnaire (FFQ) developed by Walter C. Willett. This FFQ has been extensively validated and standardized in several multiethnic population-based prospective and cross-sectional studies and for determination of chronic diseases such as CVD and T2D [15,16]. Participants reported their consumption of various foods over the past year. This FFQ describes individuals’ intake of macro- and micronutrients, dietary patterns, food habits, changes of consumption in last 10 years, frequency of foods, usual serving size, and servings per week of food items that are not listed. The FFQ has several advantages: it is self-administered, inexpensive, subject burden is minimal, and does not require specialized training for the participant.

#### Statistical analysis:

Prior to analysis, all continuous variables were tested by Q-Q plots and when needed were log-transformed to achieve linearity. Total n-3 intake (from ALA, DHA, and EPA in food and supplements) was transformed into a binary variable for adequate versus inadequate intake in grams/day. Since no definitive recommendations are given for n-3 cut-offs were considered as adequate for either 1 g or 2 g per day based on model fit. Participants were excluded if they met one or more of the following conditions: 1) missing values n-3, HCY, or hs-CRP; 2) CRP higher than 10 mg/L (n=95) since this elevation might be a result of an acute infection and cannot be used to asses CVD risk [17]; and 3) total energy intake <500 or >5000 kcal/day. A total of 490 participants (HA=238; AA=172) with and without type 2 diabetes were included in the final data analysis. Logistic regression models were used to assess the odds ratios for n-3 intake. One and two-way interactions were tested for ethnicity and diabetes status with n-3. Analysis was conducted for each ethnicity, based on the significance of the two-way interactions. Adjustment variables for final models included: age, gender, smoking status, waist circumference, and Kcal/day. Covariates vitamin B, saturated fat, fiber, and physical activity were tested along with the adjustment variables and not retained. A binary variables were created for HCY, where the 75^th^ percentile and above was considered high (12 mg/L) and for CRP, where high CRP was considered ≥ 3 mg/dL. Data were analyzed using the statistical software IBM SPSS Statistics version 19 (SPSS Inc, Chicago, IL) and statistical significance was set at p<0. 05.

## Results

General characteristics of the study population by ethnicity are presented in table 1. There was no difference in the gender distribution between AA and HA (p=0.327); albeit, AA were approximately 3 years younger than HA. African Americans consumed more calories and higher grams of n-3; however, n-3 per kilocalorie was not statistically different between them (AA=0.859×10^−4^; HA=0.847×10^−4^). African Americans had higher waist circumferences (p<0.001) and CRP (p<0.001) as compared to HA; but there was no statistical difference between ethnicities in HCY levels (p=0.714).

Table 2 depicts the logistic regression model for the odds of having high CRP (≥ 3 mg/L) by n-3 consumption, ethnicity and diabetes status. Low n-3 intake (<2 g/day) was not an independent predictor of CRP as hypothesized. Instead, ethnicity by n-3 intake was significant. African Americans with low n-3 intake were more likely to have high CRP as compared to their counterparts [OR=3.21 (1.11, 9.26) p=0.032]. This relationship was not significant for HA (data not shown). Although having diabetes was associated with high CRP, as expected, diabetes status by n-3 intake was not a significant predictor of high CRP (p=0.132).

The logistic regression model for the odds of high HCY (>12 mg/L) by n-3 intake, ethnicity and diabetes status is presented in table 3. African Americans with low n-3 intake (<1 g/d) were more than four times likely to have high HCY as compared to their counterparts [OR=4.36 (1.59, 12.0) p=0.004]. Again, this relationship was not significant for Haitian Americans (data not shown). Diabetes status and diabetes by n-3 intake were not associated with HCY in this model. A comparison of means was conducted for each ethnicity for HCY levels and there were no differences by diabetes status for AA (p=0.235) and for HA (p=0.412) (data not shown).

## Discussion

This study investigated n-3 and its association with lipid profiles, CRP and HCY in AA, and HA with and without T2D. Ethnicity modified the relationship between omega 3 intake and the cardiovascular risk markers, CRP and HCY. Most studies for n-3 intake with either CRP or HCY are performed in specific populations, but do not compare across ethnicities and diabetes status. Moreover, the association among n-3 and CRP levels in cross sectional studies and the effects of omega-3 supplementation/fish intake on serum CRP levels have inconsistent findings. Similarly, n-3 interventions have produced inconclusive results for HCY due to small sample sizes and short duration of supplementation.

### Effect of omega 3 on CRP

Contradictory findings have been found for Caucasian populations. Several intervention studies have shown improvements in CRP corresponding to n-3 consumption among Caucasian populations [18–20]. On the other hand, no improvements in CRP were found after a placebo-control intervention with n-3 in a small sample of healthy, middle-aged, Caucasian adults [21]. Conversely, an inverse relationship between n-3 intake and CRP level was found for a large sample of Caucasian men, aged 42–60 years [22]. A cross-sectional study of healthy Australian adults showed an inverse association of CRP with n-3 intake [23]. N-3 levels assessed in blood were inversely associated with CRP in a group of healthy adults [24]. Tumor necrosis factor (TNF-α), another indicator of inflammation, but not CRP, was lowered after n-3 supplementation (2.4 g/day) for a small group of hemodialysis patients [25]. C-reactive protein levels reduced after a 12-week intervention for the group given n-3 and statins as compared to the control given statins only in patients with dyslipidemia [26,27]. N-3 consumption in the form of non-fried fish was associated with lower CRP levels in a cross-section study of the multi-ethnic study of atherosclerosis (MESA) cohort [28].

The metabolic role of n-3 as a supplement for individuals with type 2 diabetes has a matter of debate due to a potential for increase in hemoglobin A1c, fasting blood glucose, and lipid peroxidation [28,29]. Shidfar et al. [29] found no effects on glycemic control or lipoproteins with a supplementation of 2 g per day of n-3 and attribute disparate findings to differences in diabetes medications, the presence of insulin resistance, hypertension, and obesity. Evidence for the reduction of CRP by n-3 in populations with type 2 diabetes has not been established. Several intervention studies showed no effect of n-3 supplementation on CRP for populations with type 2 diabetes [28,30]. C-reactive protein levels were not affected by n-3 supplementation (3 g/day for eight weeks) in a randomized control trial for persons with type 2 diabetes; yet IL-2 and TNF-α, other indicators of inflammation, were reduced in the n-3 group [30]. No significant changes in CRP were found after two-month supplementation with n-3 for individuals with type 2 diabetes; however, HCY levels were significantly reduced [28]. A meta-analysis of n-3 and its effect on inflammatory factors from earlier studies found only two trials evaluating CRP on individuals with type 2 diabetes [31]. N-3 did not significantly lower CRP for either trial [31]. However, n-3 in blood was inversely associated with CRP in obese and non-obese, otherwise healthy, Greek adults [32]. These studies suggest that low-grade, systemic inflammation, measured by CRP, is a chronic disease indicative of life-long diet and may not be responsive to short-term intervention.

### Effect of n-3 with HCY

Supplementation with n-3 was associated with a decrease in HCY according to a meta-analysis of 11 intervention trials [33]. N-3 supplementation was associated with the reduction of HCY for healthy individuals [18], women [34], and for adults with type 2 diabetes and dyslipidemia [35]. Several n-3 supplementation trial aimed to lower HCY contained other treatments such as statins, fiber, or antioxidant-rich juices. Reduction in HCY after 12-weeks with n-3 supplementation (3.6 g/day) for otherwise healthy individuals with elevated HCY also included statins, multivitamins and fiber; however, there was no reduction in HCY in the placebo group (statins, only) [18]. Tomato juice enriched with n-3 (250 mg EPA+DHA) significantly reduced HCY (approximately 3 μM), after 2-weeks, as compared to tomato juice without n-3 for a small sample of healthy women; however, by the authors’ own admission, both juices provided antioxidant compounds such as phenolics and lycopene [34].

Similarly, cross-sectional studies have shown high n-3 intake associated with low HCY for patients with type 2 diabetes [28], healthy males [36] and older Chinese adults [37]. The protective effect of n-3 may be attributed, in part, to its role in activating other antioxidants. In fact, n-3 has been associated with increased activity of glutathione peroxidase, an antioxidant enzyme with selenium acting as a cofactor, in a population with type 2 diabetes [38].

### Confounders of n-3 with CRP and HCY: metabolic state, ethnicity, genetics

It has not been determined whether ethnic differences in the relationships between either CRP or HCY can be attributed to metabolic, cultural, or genetic factors. With respect to metabolic differences, there is an increasing number of individuals, particularly non-Caucasian ancestry, that are of a presumable ‘normal body weight’, but have a phenotype that can be referred to ‘metabolically obese’ [39]. Increased susceptibility to CVD and diabetes may be due, in part, to high free-radical level in plasma which impairs insulin function [40]. The association of omega 3 intake with HCY and CRP for AA in the present study may be due, in part, to their susceptibility to cardiovascular disease; however, prospective studies are needed to make any determinations. Another possible confounder is type of n-3 (EPA/DHA versus ALA) and its interaction with metabolic factors. Metabolic effect differences were found between fish (a source of EPA/DHA) and flaxseed oil (a source of ALA) on glucose homeostasis, but not on CRP or lipid metabolism in a primarily Caucasian cohort of women with polycystic ovary syndrome [41]. Although there were several ethnicities in this 6-week prospective randomized controlled study, their numbers were insufficient to access ethnic influence on the effect of n-3 on metabolic outcomes [41]. For Caucasian patients with end-stage renal disease, serum DHA, but not EPA was inversely associated with plasma HCY [42]. The higher degree of unsaturation in DHA (22:6) as compared to EPA (22:5) may be a factor in hemodynamic and anti-atherogenic properties that affect HCY metabolism as well as the metabolic state of the target population [43]. A major cultural factor of ethnic differences, food preparation, may change the effectiveness of n-3; in particular, deep frying [44]. Ethnic difference in cooking may have influenced the relationship between n-3 with HCY and CRP. Food preparation may differ by ethnicity for our participants since AA were born in the US; whereas HA were all born in Haiti. Vast differences in diet were reported between AA and HA [45]. Finally, genetic differences among ethnicities may be a factor influencing the association of n-3 with HCY and CRP; however, there are no studies, to date, that have investigated this area. Since n-3 has been shown to regulate gene expression for antioxidant as well as reactive oxidative species, it is plausible that the genetic differences between AA and HA may explain differences in the effectiveness of n-3 in metabolic functions. The extent to which the multifaceted aspects of ethnicity: metabolic, cultural, and genetic, modify the relationship between n-3 with HCY and CRP has not been established.

### Limitations

This study had several limitations. The results were cross-sectional and causality cannot be assumed. N-3 was assessed by a food frequency questionnaire and the actual blood levels may differ. Food preparation and self-report of dietary data may have bias the results. On the other hand, a food frequency questionnaire could represent a yearly average intake of n-3; whereas, blood levels of n-3 represent recent consumption. Although other antioxidants were not correlated with CRP and HCY, they were not considered in the final models and may have some confounding effect in the relationship between n-3 consumption and CRP and HCY levels.

## Conclusion

N-3 intake was significantly associated with CRP and HCY levels for AA but not for HA. Diabetes status and consumption of n-3 did not significantly predict odds of high CRP or high HCY. These findings suggest the need for prospective and intervention studies of measurement of n-3 in blood levels and cardiovascular events follow-up between and across multiethnic populations with and without type 2 diabetes.

## Figures and Tables

**Table 1:** General characteristic by ethnicity.

Variable	African American	Haitian American	P-value
Categorical variables N (% within ethnicity)			
Gender (males)	93 (54.1)	115 (49.1)	0.327
Low n-3<1 g/day	43 (25.0)	97 (41.5)	0.001
High HCY (>12 mg/L)	45 (26.2)	54 (23.1)	0.474
High CRP (≥ 3 mg/L)	76 (44.2)	64 (27.4)	0.001
Current smoker	62 (36.0)	15 (6.4)	<0.001
Continuous variables (mean ± SD)			
Age (years)	53.3 ± 9.5	56.0 ± 11	0.008
Waist circumference (cm)	106.7 ± 17	97.9 ± 12	<0.001
Homocysteine (mg/L)	10.3 ± 4.0	10.2 ± 3.8	0.714
CRP (mg/L)	3.15 ± 2.5	2.21 ± 2.0	<0.001
N-3 (grams)	1.89 ± 1.3	1.49 ± 1.1	0.001
In Kcal	7.57 ± .51	7.34 ± .51	<0.001
Kcal	2199 ± 1043	1759 ± 942	<0.001

**Abbreviations:** HCY=homocysteine; CRP=high-sensitivity C=reactive protein; n-3=omega-3 fatty acids.

**Table 2:** Odds ratio of high C-reactive protein (CRP) (≥ 3 mg/L) by omega-3 fatty acid intake, ethnicity and diabetes status.

Variable		95% C.I for OR		p value
	OR	Lower	Upper	
African American	0.57	0.22	1.49	0.249
Haitian American (reference)	1.00	1.00	1.00	-
African American by low omega-3 fatty acids	3.21	1.11	9.26	0.031
low omega-3 fatty acids<2 g/day	1.38	0.541	3.51	0.502
With type 2 diabetes	2.72	1.08	6.82	0.033
With diabetes by low omega 3	0.44	0.155	1.28	0.132

Model Summary: χ^2^(10)=67.1 N=406 p<0.001

Nagelkerke R^2^=0.210.

The model classified 74% of the cases of CRP, correctly.

Model adjusted by kcal (p=0.307), waist circumference (p<0.001), gender (p=0.262), smoking (p=0.587), and age (p=0.510).

**Table 3:** Odds ratio of high homocysteine (HCY) (>12 mg/L) by omega-3 fatty acid intake, ethnicity and diabetes status.

	OR	95% C.I. for OR		
Variable		Lower	Upper	p value
AA by low omega-3 fatty acids (≤ 1 g/day)	4.36	1.59	12.0	0.004
African American	0.61	0.31	1.20	0.155
Haitian American (reference)	1.00	-	-	-
Diabetes by low omega-3 fatty acids	1.02	0.36	2.90	0.965
Diabetes	1.10	0.59	2.03	0.770
Low omega-3 fatty acids (≤ 1g/day)	0.75	0.58	2.02	0.576

Model Summary: χ^2^ (10) (N=406)=30.9; p=0.001.

Nagelkerke R^2^=0.109. The model classified 75% of the cases of HCY, correctly.

The model was adjusted by age (p=0.001), gender (p=0.305), waist circumference (p=0.225), smoking (p=0.029), and calories (p=0.765).
